# Prenatal Hypoxia Is Associated with Long-Term Retinal Dysfunction in Rats

**DOI:** 10.1371/journal.pone.0061861

**Published:** 2013-04-16

**Authors:** Stephane L. Bourque, Sharee Kuny, Laura M. Reyes, Sandra T. Davidge, Yves Sauvé

**Affiliations:** 1 Department of Obstetrics and Gynecology, University of Alberta, Edmonton, Alberta, Canada; 2 Department of Ophthalmology, University of Alberta, Edmonton, Alberta, Canada; 3 Department of Physiology, University of Alberta, Edmonton, Alberta, Canada; 4 Women and Children's Health Research Institute, Edmonton, Alberta, Canada; Dalhousie University, Canada

## Abstract

**Background:**

Intra-uterine growth restriction (IUGR) has been associated with increased predisposition to age-related complications. We tested the hypothesis that rat offspring models of IUGR would exhibit exacerbated, age-related retinal dysfunction.

**Methods:**

Female Sprague-Dawley rats (maintained at 11.5% O_2_ from gestational day 15 to 21 to induce IUGR) and control offspring (maintained at 21% O_2_ throughout pregnancy) had retinal function assessed at 2 months (young) and 14 months of age (aged) with electroretinogram (ERG) recordings. Retinal anatomy was assessed by immunofluorescence.

**Results:**

Deficits in rod-driven retina function were observed in aged IUGR offspring, as evidenced by reduced amplitudes of dark-adapted mixed a-wave V_max_ (by 49.3%, P<0.01), b-wave V_max_ (by 42.1%, P<0.001) and dark-adapted peak oscillatory potentials (by 42.3%, P<0.01). In contrast to the rod-driven defects specific to aged IUGR offspring, light adapted ERG recordings revealed cone defects in young animals, that were stationary until old age. At 2 months, IUGR offspring had amplitude reductions for both b-wave (V_max_ by 46%, P<0.01) and peak oscillatory potential (V_max_ by 38%, P<0.05). Finally, defects in cone-driven responses were further confirmed by reduced maximal photopic flicker amplitudes at 2 (by 42%, P<0.001) and 14 months (by 34%, P = 0.06) and critical flicker fusion frequencies at 14 months (Control: 42±1 Hz, IUGR: 35±2 Hz, P<0.05). These functional changes were not paralleled by anatomical losses in IUGR offspring retinas.

**Conclusions:**

These data support that the developing retina is sensitive to stressors, and that pathways governing cone- and rod-driven function differ in their susceptibilities. In the case of prenatal hypoxia, cone- and rod-driven dysfunction manifest at young and old ages, respectively. We must, therefore, take into account the specific impact that fetal programming might exert on age-related retinal dystrophies when considering related diagnoses and therapeutic applications.

## Introduction

Numerous lines of evidence support that fetal hypoxia is an important etiological factor in a number of pregnancy-related complications such as preeclampsia and intrauterine growth restriction (IUGR). In the last two decades, epidemiological and animal studies have shown that *in utero* stressors can alter developmental trajectories in the fetus; thereby increasing its propensity to long-term health complications [1]. Neural networks, including sensory systems such as vision, may be particularly prone to insults during pregnancy due to the fact that their development and maturation is highly time and context dependent. In humans, a suboptimal uterine environment has been associated with alterations in color perception, acuity and contrast sensitivity [2]–[4]. More recently, studies in rodents have shown that intrauterine compromise, secondary to chronic placental insufficiency, causes altered retinal structure and function [5]–[7]. However, in these aforementioned studies, the effects of a suboptimal uterine environment were studied exclusively in young offspring, and consequently long-term effects have not been studied.

Normal aging has a well-established effect on retina and is associated with alterations in various aspects of visual information processing (reviewed in [8]). Even in the absence of overt pathology (e.g. macular degeneration or glaucoma), the retina undergoes age-related changes, typically characterized first by rod dysfunction, then followed by cone dysfunction [9], resulting initially in delayed dark-adaptation as assessed both psychophysically [10] and with eletroretinogram (ERG) recording [11], [12]. Given the steadily increasing percentage of the population over the age of 60 in many countries worldwide, age-related vision disorders are predicted to increase in tandem [13]. Consequently, understanding the mechanisms that govern changes in retinal function with age is crucial and timely. Indeed, identifying factors that predispose individuals to early onset or more severe age-related disorders of the eye could be of considerable advantage in targeting susceptible individuals or populations for early intervention.

In this study, we tested the hypothesis that offspring of dams exposed to hypoxia during pregnancy would exhibit aggravated age-related changes in retinal structure and function. The findings obtained herein suggest that prenatal hypoxia does cause retinal defects that present at a young age (cone-driven), as well as defects that manifest in later life (rod-driven). Furthermore, we demonstrate that these deficiencies stem from physiological perturbations in retinal function, as opposed to anatomical loss.

## Materials and Methods

### Ethics Statement

The experimental protocols described herein were conducted in accordance with the guidelines established by the Canadian Council for Animal Care, and were approved by the University of Alberta Animal Care and Use Committee (License #463). The investigations conform to the *Guide for the Care and Use of Laboratory Animals* published by the US National Institutes of Health (NIH Publication No. 85-23, revised 1996).

### Animals and treatments

Prenatal hypoxia was induced as previously described [14]. Briefly, female Sprague Dawley rats were purchased from Charles River (Wilmingham MA) and housed in the University of Alberta Animal Care Facility. Rats had *ad libitum* access to food and water. After one-week acclimatization, dams were bred with age-matched males and vaginal smears were checked daily for the presence of sperm; the presence of sperm was considered gestational day (GD)0 [15]. At GD15, pregnant dams were randomized to either the control or IUGR group. Rats in the IUGR group were housed individually in a standard cage inside a plexiglass chamber, and the environmental oxygen partial pressure (PO_2_) was reduced to 11±0.2% using a regulated infusion of nitrogen gas. Oxygen levels were monitored continuously throughout the treatment period using an oxygen analyzer (Hudson RCI, Temecula, CA). Control animals were housed individually in a room air (21% PO_2_) environment. At GD21, rats were removed from the hypoxic chamber, and allowed to give birth in a normal oxygen environment. This treatment regimen has been shown to cause fetal hypoxia and IUGR [16]. At birth, litters were reduced to 8 offspring to standardize postnatal conditions. At postnatal day 21, offspring were weaned onto standard rat chow, and maintained in the colony until day of testing.

### ERG recordings and analyses

At 2 months and 14 months of age, retina function was assessed in control and IUGR rats (n = 6 each per time point), by recording the full-field ERG as previously described [17]. In brief, following one-hour dark-adaptation, animals were prepared for ERG recordings under dim red light. Animals were anesthetized i.p. with ketamine (75 mg/kg) and xylazine (15 mg/kg), placed in prone position, and body temperature maintained at 38°C with a homeothermic blanket. Pupils were dilated with a topical solution of 1% tropicamide. Bilateral recording of ERG responses (amplified at 0.3–300 Hz, without 60 Hz notch filtering; Espion E^2^ system from Diagnosys LLC, Lowell MA) was achieved with gold loop active electrodes placed on each cornea and platinum reference electrodes inserted subdermally behind each eye; a ground platinum electrode was inserted subdermally in the scruff. Stimuli consisted of white flashes generated by a xenon bulb (6500°K color temperature, 10 µs duration) unless otherwise noted. Calibration of light levels was achieved using an IL1700 photometer (International Light Technologies Inc., Peabody MA) equipped with either a photopic or scotopic filter. Firstly, dark-adapted intensity responses (stimuli presented at 19 increasing intensities varying from −5.7 to 2.9 log cd·s/m^2^; logarithm of scotopic candela seconds/meter square) were recorded. Dark adaptation was used to assess rod-driven function. Secondly, we recorded light-adapted (30 cd/m^2^ white light background) photopic intensity responses (stimuli presented at 11 increasing intensities varying from −1.63, to 2.86 log cd·s/m^2^), followed by flicker responses (stimuli of 1 log cd·s/m^2^ luminance presented at 11 increasing frequencies varying from 3 to 60 Hz). Light adaptation was used to saturate rod photoreceptors and therefore isolate pure cone-driven responses.

ERG recordings from one eye per animal were analyzed; the eye chosen in each animal was the one in which the saturated dark-adapted a-wave had the highest amplitude. Criterion amplitudes were set at 10 µV for a-, b-waves and flicker peak-to-trough responses; a magnitude below which signals could not be clearly distinguished from background noise. The amplitude of the b-wave was measured as the difference from the negative a-wave trough to the peak of the b-wave and not the oscillatory potentials (OP). The b/a amplitude ratios were calculated only when both a- and b-waves exceeded the 10 µV amplitude criterion. Isolated OP traces were generated using a 75–300 Hz digital filter in the Espion E^2^ system. For quantification, peak OP amplitudes were obtained using Morlet wavelet transform [18]. Finally, critical flicker-fusion frequency (CFF) corresponded to the lowest frequency for which peak-to-trough responses did not exceed a 10 µV criterion amplitude.

### Immunofluorescence microscopy

IUGR and control age-matched offspring retinas were studied immunohistochemically at 2 months and 14 months of age (n = 3 each IUGR and control at both time points). Retina cross-sections were prepared as previously reported [19], [20]. Briefly, after lens removal, eyes were post-fixed for 30 minutes at room temperature in 4% paraformaldehyde (pH 7.4) and cryoprotected in graded sucrose concentrations (10% for 1 h, 20% for 1 h, and 30% overnight) at 4°C. Tissues were placed in molds (Tissue Path disposable base molds 7×7×5 mm, Fisher Scientific, Ontario, Canada) filled with embedding medium (Tissue-Tek O.C.T. Compound; Sakura Finetek USA, Inc., Torrance, CA), flash frozen in liquid nitrogen, and stored at −80°C until sectioned. After embedding, 20 µm cross-sections were cut and mounted on glass slides (Superfrost Plus; Thermo Fisher Scientific,Waltham, MA). After hydration in PBS, the sections were blocked for 1 hour in PBS +0.3% Triton X-100 +10% serum (same species as secondary antibody), and reacted overnight in a humid container with the following primary antibodies: mouse anti-rhodopsin (clone 4D2, Millipore, MABN15, 1∶500); goat anti-OPN1SW (N-20, Santa Cruz Biotech, sc-14363, 1∶500) in combination with rabbit anti-M/L-opsin (Millipore, AB5405, 1∶500); rabbit-PKCalpha (C-20, Santa Cruz Biotech, sc-208, 1∶200) in combination with mouse anti-bassoon (Stressgen, VAM-PS003, 1∶250); mouse anti-glial fibrillary acidic protein (GFAP ; Covance, SMI-22R, 1∶1000); goat anti-ChAT (Millipore, AB144P, 1∶200); and rabbit anti-TH (Millipore, AB152, 1∶500). The following day, after extensive washing in PBS, sections were reacted for 1 hour with species-appropriate secondary antibodies conjugated to fluorescent dyes (1∶1000, Alexa; Molecular Probes, Eugene,OR). After additional washing, slides were coated with an antifade reagent with DAPI (Prolong Gold, Molecular Probes, P36931) and coverslipped. All antibodies were diluted in a 1∶10 solution of the blocking medium and all incubations were performed at room temperature. Images were captured from the center of the retina (400±200 µm from the edge of the optic nerve) using either a confocal microscope (Zeiss LSM710, with a Plan-Apochromat 40×/1.3 oil objective) or a fluorescent microscope (Leica DMRE6000B, 20× objective). This region, 200–600 µm from the edge of the optic disk was chosen for to reasons: 1) to ensure eccentricity was similar for all animals, therefore allowing for direct comparisons; 2) to avoid any artifactual differences in thickness due to cutting angle. Images were projections of *z*-stacks of 9 to 12 slices of 1 µm. Brightness and contrast levels were adjusted if necessary (Adobe Photoshop CS2 software version 9.0.2; Adobe, San Jose, CA).

### Statistical Analyses

Data are presented as mean±SEM. Data were analyzed by 2-way ANOVA, for the effects of age and prenatal treatment; where overall significance was found, data were subsequently analyzed using Bonferonni post-hoc test. In the case of photopic a-wave amplitudes, which under many circumstances did not achieve criterion amplitudes, these data were analyzed using chi-squared test.

## Results

### Dark-adapted retina function

**Figure 1:**
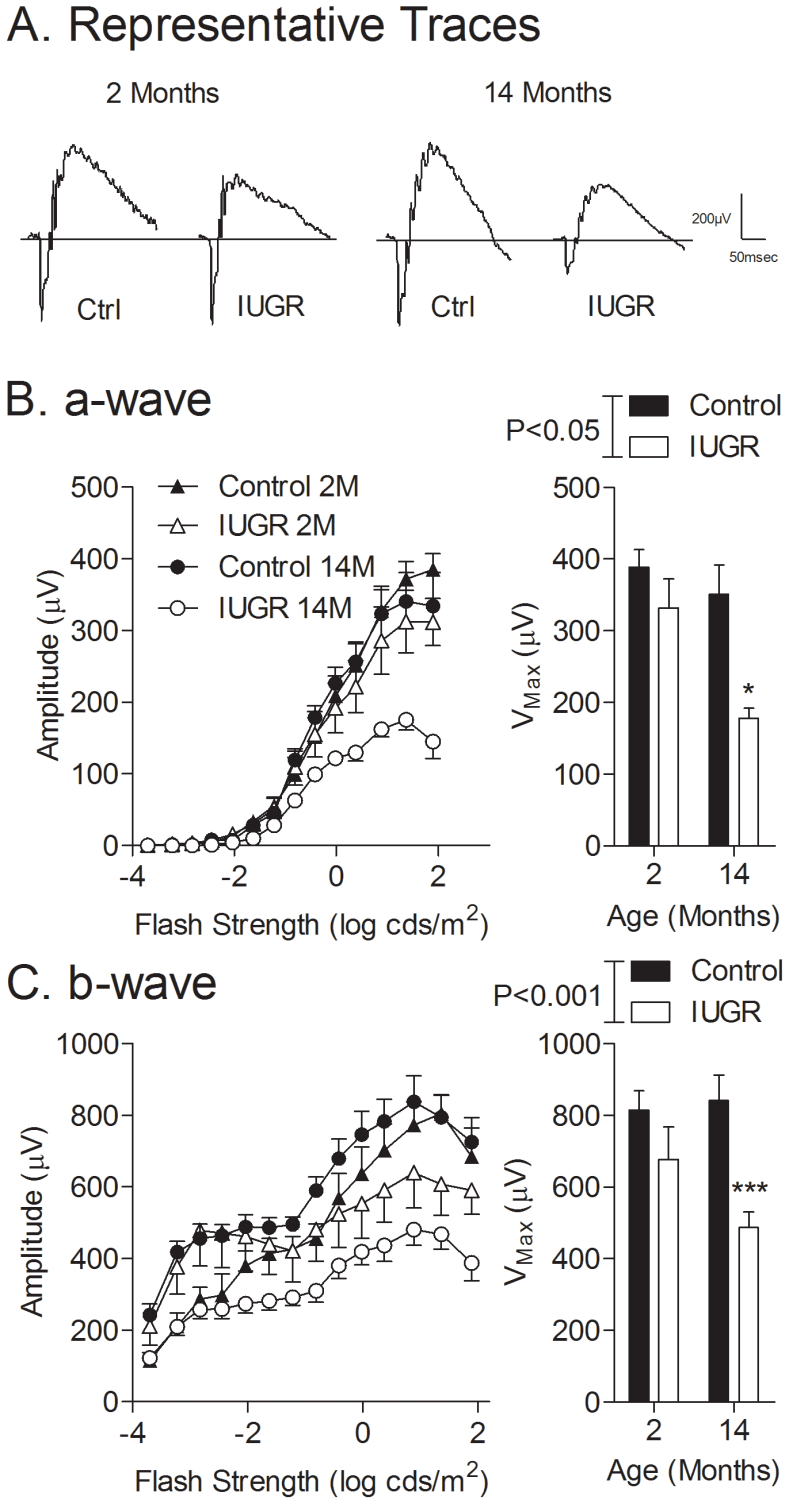
Dark-adapted ERG a-wave (photoreceptoral) and b-wave (post-photoreceptoral) elicited by step-wise increases in stimulus strength. (A) Representative traces corresponding to responses elicited by a stimulus intensity of 1.36 log cds/m2; (B) a-wave, and (C) b-wave amplitudes measured in control and IUGR offspring at 2 and 14 months of age. Adjacent bar graphs depict corresponding peak amplitudes. **P<0.01 compared to controls at the same age.

Dark-adapted a- and b-wave amplitudes are shown in Figure 1 (representative traces are presented in panel A, and summarized data are presented in panels B and C). In all animals studied, dark-adapted a-wave amplitudes increased (Figure 1B) and implicit times decreased (graphs not shown) proportional to stimulus intensity. Compared with controls, offspring exposed to prenatal hypoxia (IUGR group) had a-wave amplitudes reduced by 49.3%, (P<0.01) at 14 months (V_max_; Control: 351.2±40.7 µV, IUGR: 178.1±13.75 µV; P<0.05), but not at 2 months of age (Figure 1A and 1B). This effect was most apparent at high stimulus intensities (associated with maximal a-wave amplitudes), which were reduced by almost 50% in aged IUGR offspring compared to age-matched controls. While a-wave implicit times were prolonged in IUGR compared to controls (biggest trend at 0.3 log cd·s/m^2^), statistical analysis using 2-way ANOVA showed no significant effect (Table 1).

**Table 1:** Minimum recorded implicit times (IT) in young (2 month) and aged (14 month) offspring.

	2 Months		14 Months		2-WAY ANOVA OUTCOMES		
	Control	IUGR	Control	IUGR	IUGR	Age	Int.
**Scotopic**							
a-wave IT (ms)	6.9±0.6	5.6±0.3	6.5±0.1	6.4±0.4	0.07	0.53	0.14
b-wave IT (ms)	59.8±7.1	51.6±8.2	66.3±3.9	67.1±4.8	0.52	0.07	0.43
**Photopic**							
b-wave IT (ms)	30.7±3.1	37.4±1.3	59.0±5.5	65.0±9.5	0.28	0.96	0.22

Photopic a-wave implicit times are not shown because of the lack of complete data sets in each group; notably, 14 month IUGR offspring had only one rat achieve criterion responses (see Figure 3B).

The relationship between b-wave amplitude and stimulus intensities (Figure 1C) was similar to that for a-wave amplitude with the exception that the curve was biphasic; with a first plateau preceding the threshold for cones (and coinciding with a-wave onset) and a second plateau at which maximal amplitudes were reached (Figure 1C). Significant effects were seen for both IUGR (P<0.01) and age (P<0.01), as assessed by 2-way ANOVA; post-hoc testing revealed attenuated b-wave peak amplitudes by 42.1% (P<0.001) in aged (V_max_; Control: 841.5±71.2 µV, IUGR: 486.8±43.7 µV), but not young. In contrast to a-wave amplitudes, b-wave amplitude differences were equally evident at both low and high stimulus intensities, reflecting pure-rod and mixed rod-cone-driven responses, respectively. Beyond stimulus intensities of 0.88 log cd·s/m^2^, amplitude reductions were observed, suggesting photoreceptor saturation [21]. As observed for a-waves, b-wave implicit times decreased proportionally to stimulus intensity, reaching a plateau of fastest implicit times at higher stimulus intensities (beginning at 1.62 log cd·s/m^2^); however there were no differences between groups in b-wave implicit times (Table 1).

**Figure 2:**
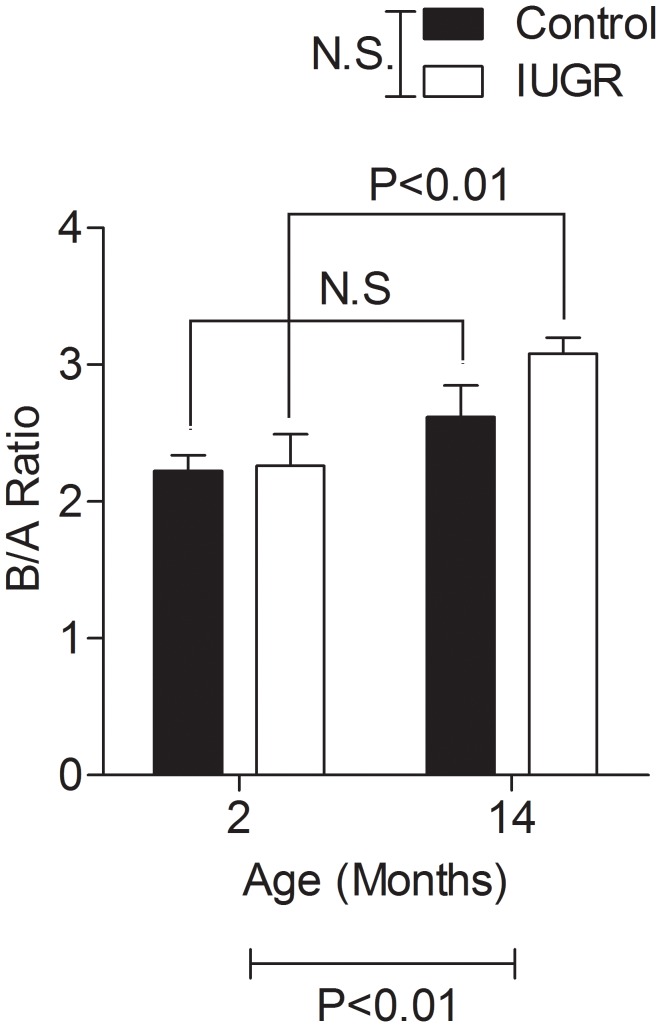
Dark-adapted b/a ratios as a function of age in control and IUGR offspring. No differences were seen between groups at either 2 or 14 months of age. However, the b/a ratio increased in the IUGR group between 2 and 14 months (P<0.01).

To assess potential postsynaptic compensation, as previously postulated with age in humans [12], b/a amplitude ratios were calculated at the stimulus intensity yielding the highest a-wave amplitudes (Figure 2). By 2-way ANOVA, we detected no overall effect of prenatal treatment on b/a ratios (Figure 2), although there was an overall effect of age (P<0.01); posthoc analysis revealed that this difference was only apparent in the IUGR group.

### Light-adapted retina function

**Figure 3:**
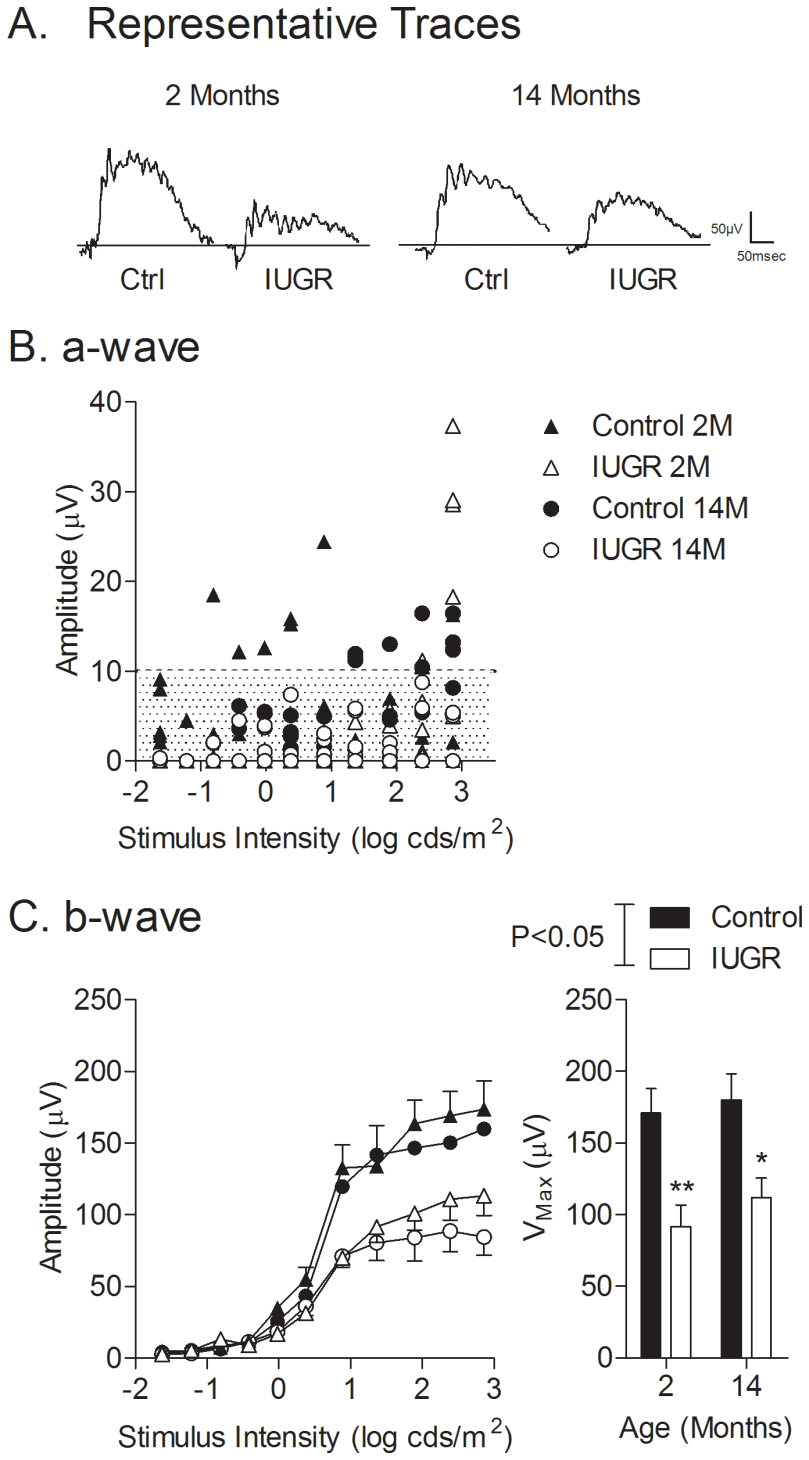
Light-adapted ERG a-wave (photoreceptoral) and b-wave (post-photoreceptoral) elicited by step-wise increases in stimulus intensities. (A) Representative traces corresponding to responses elicited by a stimulus intensity of 1.36 log cds/m^2^; (B) a-wave, and (C) b-wave amplitudes measured in control and IUGR offspring at 2 and 14 months of age. A-wave data (B) depict responses of each rat tested rather than means because the majority of responses were below the threshold criteria of 10 µV (shaded area). *P<0.05, *P<0.01 compared to controls at the same age.

Figure 3A shows representative traces of light-adapted ERG recordings in controls and IUGR at 2 months and 14 months of age. A- and b-wave amplitudes under light-adapted conditions are shown in Figure 3. For flash intensities inferior to the 1.36 log cd·s/m^2^ threshold, a-wave amplitudes were largely below the 10 µV criterion threshold (Figure 3B) as expected. In young, 5 out of 6 offspring in each group achieved threshold responses. In old, 4 out of the 6 control offspring exceeded the criterion amplitude, compared to none of the 6 IUGR offspring studied (P = 0.01, chi squared test). B-wave amplitudes were also markedly reduced in young and aged IUGR offspring (46% and 38% at 2 and 14 months, respectively) (V_max_ at 2 months; control: 170.9±16.9 µV, IUGR: 91.7±14.8 µV; P<0.01; V_max_ at 14 months; control: 179.8±18 µV, IUGR: 112.0±13.5 µV; P<0.05), without an overall effect of aging (Figure 3C). Neither a- nor b-wave implicit times were different between groups (data not shown). Due to the fact that, with the exception of 4 control offspring, light-adapted a-wave amplitudes did not exceed criterion amplitudes, corresponding b/a ratio could not be used to compare both groups.

### Oscillatory potentials

**Figure 4:**
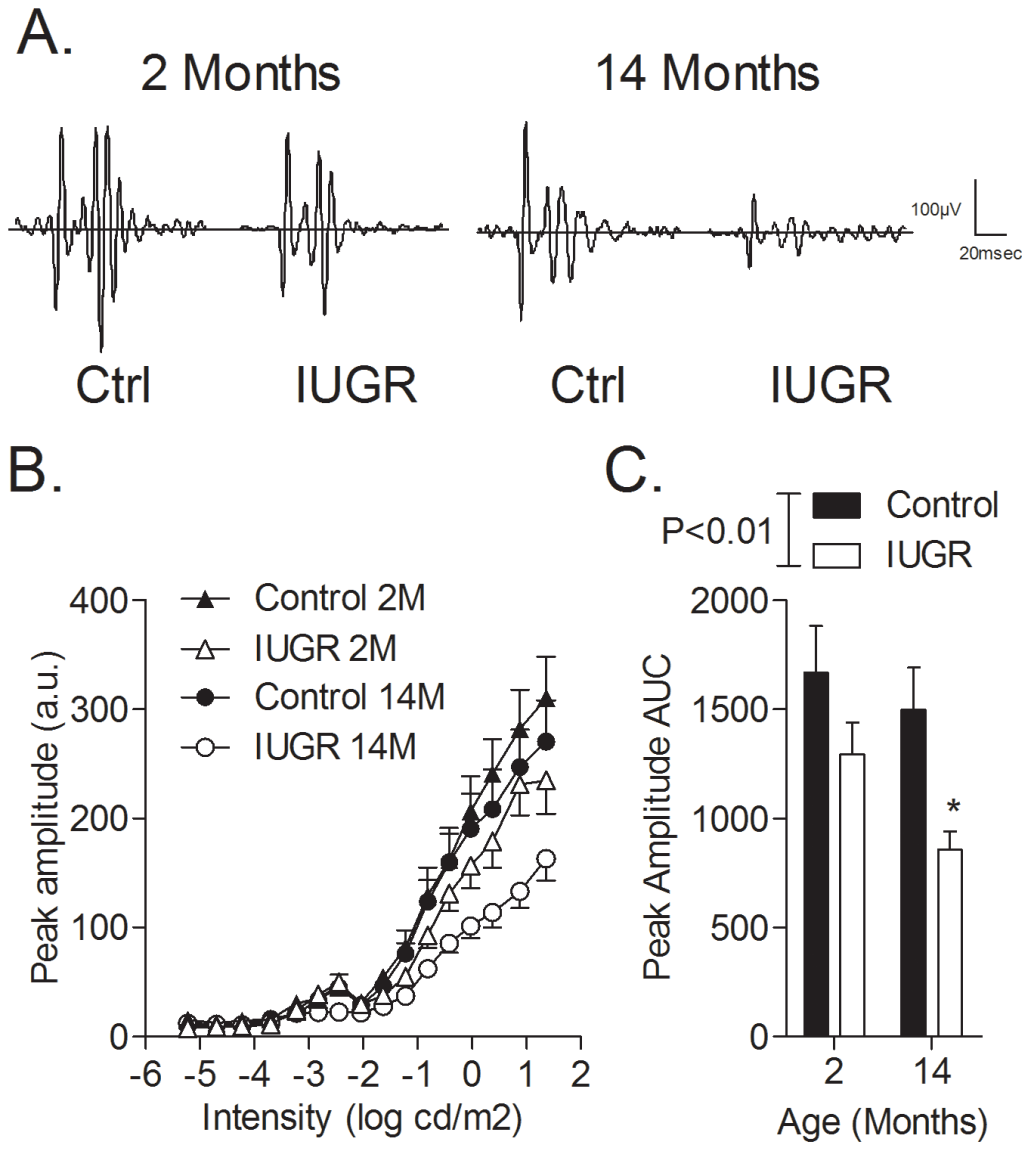
OP (post-photoreceptoral) peak amplitudes elicited by step-wise increases in stimulus intensity. (A) Representative traces corresponding to responses elicited by a stimulus intensity of 1.36 log cds/m2; (B) OP peak amplitudes measured in control and IUGR offspring at 2 and 14 months of age as a function of stimulus intensity, and (C) Bar graphs depicting comparisons of peak amplitudes of OPs elicited by the maximal stimulus intensity. *P<0.05 compared to controls at the same age. A.u., arbitrary units; AUC, area under the curve.

OP peak amplitudes under dark-adapted conditions are shown in Figure 4 (representative tracings shown in Figure 4A, and summarized data shown in Figure 4B). There was a significant effect of IUGR on peak amplitude, as assessed by comparing area under the curve (Control: 1499.5±474.2; IUGR: 858.1±271.4, P<0.01; (; Figure 4C); post-hoc testing revealed amplitude reductions of 42.3% (P<0.01), in the aged IUGR, with no differences in the young IUGR group. No changes were observed in peak times or peak frequencies between control and IUGR offspring in either dark- or light-adapted conditions (data not shown).

### Flicker Response

**Figure 5:**
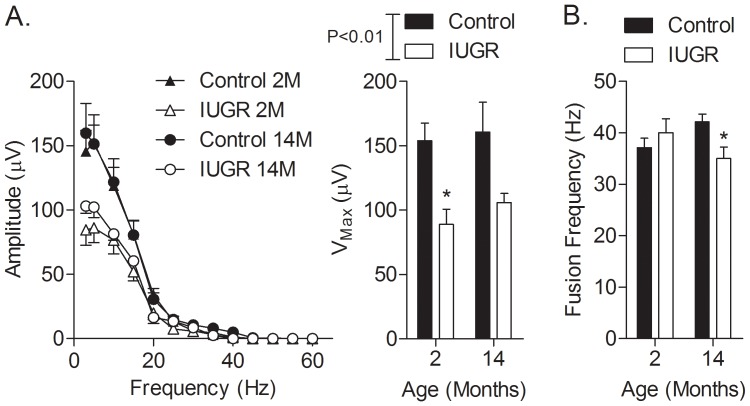
Light-adapted flicker responses (post-photoreceptoral origin). Compared to age-matched controls, response amplitude elicited by step-wise increments in stimulus frequency were reduced in IUGR offspring at both 2 and 14 months (A); *P<0.05. Compared to age-matched controls, CFF in the IUGR group, were lower at 14 months (B); *P<0.05.

Results from light-adapted flicker responses are shown in Figure 5. IUGR had lower maximal response amplitudes compared to age-matched controls at 2 months (Control: 154.0±14.7 µV; IUGR: 122.9±11.8 µV, P<0.05) and 14 months of age (Control: 160.7±23.2 µV; IUGR: 105.9±7.1 µV, P = 0.06; Figure 5A). Furthermore, CFFs were reduced in IUGR offspring at 14 months of age (Control: 42±1 Hz, IUGR: 35±2 Hz, P<0.05; Figure 5B).

### Retina anatomy

**Figure 6:**
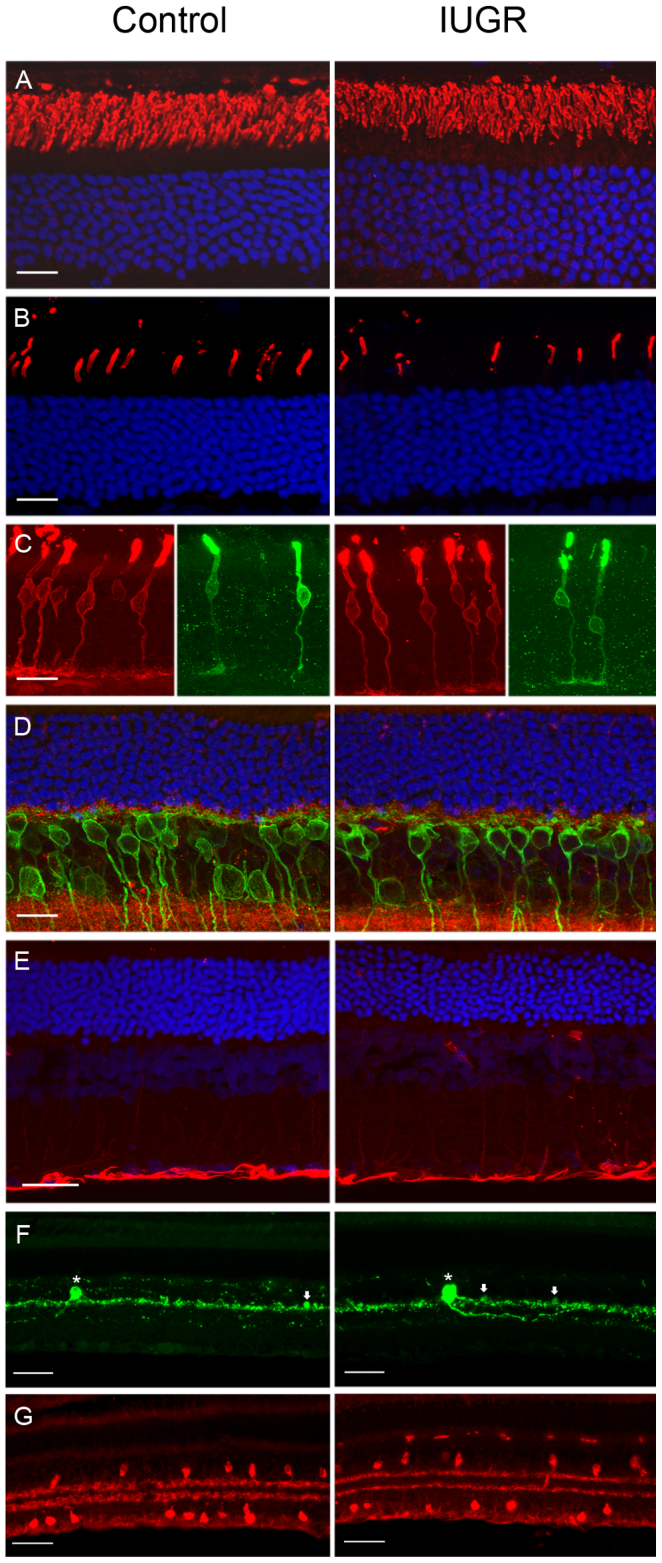
Absence of anatomical changes in the IUGR rat retina at 14 months of age. (A) Rhodopsin staining of rod photoreceptor outer segments. (B) m-Opsin staining of cone outer segments. (C) m-Opsin (r*ed*) and s-Opsin (*green*) staining of cone cell membranes. (D) PKCalpha staining of rod bipolar cells (and a subset of amacrine cells) (*green*) and bassoon staining of rod and cone ribbon synapses (*red*). (E) GFAP labelling of astrocytes and Müller cell processes. Nuclei stained with DAPI (*blue*). Scale bar, 20 µm for panels A–D, 50 µm for panel E. (F) TH staining (*green*) of low density dopaminergic A18 amacrine cells showing both cell bodies of typical size (7–11 µm, *arrows*), as well as a very small percentage with large cell bodies (>15 µm, *asterisk*). (G) ChAT staining (r*ed*) of type I and II “starburst” amacrine cells; non-displaced, located at proximal border of INL; and displaced, located in GCL. Scale bar 50 µm for panels F, G.

No differences were observed in the number of rod or cone photoreceptors (Figure 6B and 6C) between control and IUGR offspring. More specifically, photoreceptors were assessed quantitatively by counting the number of rows, consistently 8–9 rows in each group (Figure 6). Rhodopsin staining of rods revealed no differences in fluorescence intensity or in length of outer segments between groups (Figure 6B). Opsin staining of s- and m- cones, respectively, also revealed no differences between groups (Figure 6C). Additionally, bipolar cell populations were also unchanged (Figure 6D). With respect to anatomical features, the only notable difference observed was a very subtle, but consistent, increase in GFAP staining in astrocytes and Müller cells in aged IUGR offspring when compared to age-matched controls (Figure 6E). Finally, there were no obvious anatomical differences in either low density dopaminergic A18 amacrine cells (Figure 6F)or type I and II “starburst” amacrine cells (Figure 6G) between control and IUGR offspring at either age. In view of the lack of anatomical changes between IUGR and controls, only histological data from 14 month offspring are presented.

## Discussion

Early stressors are known to impact various aspects of neural function, including sensory systems. Here, we report that adult offspring, born at term but exposed to prenatal hypoxia during their last week of gestation, exhibited alterations in retinal function, characterized by (1) specific early dysfunction of the cone system as reflected by reductions in light-adapted a- and b-wave amplitudes, as well as peak OP amplitudes and (2) age related rod dysfunction as reflected by a reduction in dark-adapted a- and b-wave amplitudes. These changes occurred without anatomical loss. The present findings are consistent with studies which reported that retinal function is susceptible to uterine compromise, such as reported in the guinea pig [5], and sheep [7]. Our findings demonstrate a pattern of cone pathway dysfunction preceding that in rods , and as such provide original insight into how specific prenatal insult might affect the nature and progression of retinal deficiencies with age.

A-waves reflect light-induced hyperpolarization in photoreceptor populations [21], whereas b-waves reflect activity of ON-bipolar (depolarizing in response to light onset) cell populations [22], [23] with potential contribution from horizontal cells [24]. Therefore, a- and b-waves reflect the functional integrity of the outer (photoreceptoral) and inner (post-photoreceptoral) layers of the retina, respectively. We studied both rod (dim flash under dark-adapted conditions) and cone (under light-adapted conditions) function, and found that photoreceptor pathways exhibited marked differences in their susceptibility to prenatal hypoxia. In rods, the discernible changes in a- and b-wave amplitude between aged control and IUGR offspring, but not seen in young, are consistent with the notion that physiological perturbations elicited by prenatal stress become phenotypic when compounded with additional stressors, including normal aging. In this regard, it is of interest to determine when the onset of this dysfunction occurs, and whether it is a gradual decline in function over a long period that mirrors the progressive changes that constitute “normal aging”, or whether it occurs suddenly, precipitated by a specific combination of events. It is tempting to speculate that rod dysfunction gradually occurs over time, since minor differences, albeit not-significant, were already apparent as early as 2 months of age. Interestingly, we observed increased b/a ratios in aged IUGR offspring compared to age-matched control offspring. We have reported increased b/a ratios in aged (60+ years) compared to young (20–39 years) human subjects [12], likely reflecting post-synaptic compensation associated with aging. Our results support the possibility that prenatal hypoxia might contribute to an exacerbation of age-related changes in the offspring.

In contrast, cone-dysfunction was already evident from an early age, and persisted without progression into adulthood. The reasons for the atypical increased susceptibility of cone-related function to prenatal hypoxia compared to rod function remain to be elucidated. This observed relative susceptibility of cones might be related to their low proportion in the retina of nocturnal species, such as rats. Dysfunction in a small number of cones might induce a functional deficit, whereas for rods, dysfunction affecting only a marginal subset of cells would be largely undetected in a background of functional rods. If this is indeed the case, cone-related dysfunction due to prenatal hypoxia may be exacerbated in albino species such as Sprague Dawley rats, where cones only account for <1% of the total photoreceptor population in the retina. Interestingly, in conditions characterized by progressive retinal degeneration (e.g. retinitis pigmentosa, diabetic retinopathy, macular degeneration) photoreceptor dysfunction affects rods prior to cones [25], [26]; in fact, there are very few conditions in which this temporal sequence is reversed. It is noteworthy, however, that in these aforementioned conditions, the loss of rods, which have been shown to release trophic factors supporting cone survival and also to provide an architectural support ensuring adequate nutrient delivery and energy metabolism for the cones [27], are causally linked to cone death; as such, the temporal sequence of photoreceptor death is an important etiological factor in the progression of these diseases. In the present study, we did not observe rod or cone loss but only their dysfunction.

To gain further understanding of the processes that may be involved in the observed retina dysfunction, we examined retinal anatomy in both young (2 months) and aged (14 months) offspring. The lack of anatomical differences at either age suggests that the deficits in retina function observed reflect dysfunction of certain cell types within the retina, rather than a decline in the number of retinal cells. This is perhaps best exemplified by the marked differences between control and IUGR offspring in OP amplitudes, which are thought to originate largely from amacrine cells, without a change in the number of TH and ChAT positive cells between groups. We cannot, however, rule out the contribution of other amacrine cell populations for which we do not have immunohistochemical markers. The lack of anatomical changes in IUGR offspring seems, at first glance, at odds with the findings of Loelinger *et al.*, who reported that chronic placental insufficiency is associated with the anatomical loss of amacrine and horizontal cells [6], [7]. While numerous factors could explain this discrepancy, including the nature of the insult, timing of the insult is particularly noteworthy. Given the fact that rats are highly altricial, hypoxia induced in the last week of gestation, as in the present study, is bound to have a marked impact on retina development, when compared with precocial models such as the sheep or guinea pig. The timing of insult may also provide additional insights as to why certain cell types appear more susceptible to hypoxia than others. Rapid proliferation and differentiation of various cell types, including retinal ganglion cells (GD10 to GD19), cones (GD10 to GD17), amacrine cells (GD10 to postnatal day 0, PD0), and rods (GD17 to PD4) are occurring during the hypoxic insult in the present model [28]. The degree of overlap, as well as timing of overlap between cell development and hypoxic insult could explain the apparent susceptibility of amacrine cells and cones in this model. However, an exhaustive developmental characterization of the retina in IUGR versus control is needed to further test this hypothesis.

The lack of anatomical changes suggests that the long-term programming effects caused by prenatal hypoxia impact the transduction pathways associated with rod and cone pathways, rather than numbers of photoreceptors. The most obvious question therefore is whether the observed deficits stem from dysfunctional photoreceptors, signal transduction efficiency through bipolar or horizontal cells, or deficits originating in output cells of the retina (e.g. retinal ganglion cells); future studies will be required to elucidate the nature of this dysfunction. However, the subtle increases in GFAP staining observed in astrocytes and Muller cells is indicative of a stress response. GFAP is an intermediate filament protein, and its over-expression has been associated with ischemia [29] and retinal degeneration [30]. We have previously reported that vulnerability to ischemia-related injury varied between cell types and organ systems [16]. Although the precise mechanism underlying this susceptibility is not known, the involvement of altered cellular bioenergetics and antioxidant mechanisms may be involved in the mild Müller cell gliosis observed at 14 months of age in IUGR rats.

Finally, the CFF is the frequency at which a flickering light stimulus can no longer be resolved and appears to be completely steady to the observer. The CFF is a measure of the rate of information processing, and may have implications for processing in temporally dependent contexts. In the retina, the CFF, is related in part to phototransduction recovery. Thus, the observation that CFF is reduced in IUGR offspring compared to controls could be indicative of numerous functional deficiencies, and therefore does not provide clear insights into the mechanisms involved therein. The value of this observation may relate to its diagnostic or prognostic potential. Since assessing CFF is technically simple and inexpensive, its use may be warranted in the wake of complications in pregnancy. Indeed, since the changes in CFF were evident at 2 months of age—well before the onset of rod-related dysfunction—alterations in CFF could be prognostic of cone or eventual rod related dysfunction.

In summary, the present study offers new insights into precocious, as well as delayed, deleterious effects of prenatal stressors on the visual system. The present findings demonstrate that prenatal stressors, such as hypoxia, can have immediate consequences on sensory function, but also lasting effects that do not manifest for decades. Moreover, the magnitude of dysfunction that occurs as a consequence of prenatal hypoxia is particularly striking, and provides a clear impetus to investigate the specific mechanisms associated with the observed retinal decline, and the nature of the susceptibility of cone dysfunction to prenatal hypoxia. As such, information regarding fetal health during pregnancy and birth outcomes indicative of prenatal stress could be useful in predicting potentially debilitating retinal damage. Indeed, as the number of seniors (people over 65 years of age) steadily increases, early therapeutic intervention as a means of mitigating or preventing retinal dysfunction will undoubtedly prove more efficacious and cost effective than the alternative of treating retinal disorders at the point when irreversible damage has occurred.
